# Excitation and Circular Dichroism Spectra of (+)-(*S*,*S*)-bis(2-Methylbutyl)chalcogenides

**DOI:** 10.3390/molecules15042357

**Published:** 2010-03-31

**Authors:** Yasushi Honda, Atsushi Kurihara, Yusuke Kenmochi, Masahiko Hada

**Affiliations:** 1 Department of Chemistry, Graduate School of Science and Engineering, Tokyo Metropolitan University, 1-1 Minami-Osawa, Hachioji-shi, Tokyo 192-0397, Japan; 2 CREST, Japan Science and Technology (JST) Agency, 5 Sanbancho, Chiyoda-ku, Tokyo 102-0075, Japan

**Keywords:** excitation spectroscopy, circular dichroism (CD) spectroscopy, bis(2-methylbutyl)chalcogenide, symmetry adapted cluster-configuration interaction (SAC-CI), Boltzmann averaging, temperature dependence

## Abstract

Theoretical electronic spectra and natural circular dichroism (CD) spectra of (+)-(*S*,*S*)-bis(2-methylbutyl)chalcogenides, Ch[CH_2_CH(CH_3_)C_2_H_5_]_2_ (Ch = S, Se, and Te), were calculated by the symmetry adapted cluster (SAC) and SAC-configuration interaction (SAC-CI) methods. Whereas the calculated CD spectrum for each stable conformation itself did not reproduce the corresponding experimental one, their Boltzmann-averaged spectra showed good agreement with the experimental results. We provided the assignment for each spectral band according to our calculation results. For the telluride compound, temperature dependence of the CD spectra was experimentally observed due to variation in the Boltzmann factor, and our calculations reproduced it qualitatively. The spectral features that we could not reproduce can be attributed to triplet transitions through the spin-orbit interaction effects as well as accuracy incompleteness on the calculation conditions.

## 1. Introduction

Circular dichroism (CD) spectroscopy is caused by the differences in absorption intensity between left and right circularly polarized lights [1], and it is useful to analyze the excitation spectra. Absorption bands in CD spectra are observed at the same energy positions as bands in excitation spectra with either positive or negative intensity. This property provides auxiliary information to band assignments in the excitation spectra, and it is particular practical in the case where there are multiple states in a narrow energy range. However, studies of CD spectra may present some difficulties. First, CD spectra often exhibit complicated shapes which are difficult to analyze. Second, similar to normal excitation spectra, when the band positions are located closely to each other, it is generally difficult to determine the accurate energies of the transitions. Furthermore, conventional organic solvents prevent observation of signals for target molecules in the short-wavelength region owing to absorption of the solvents. 

Accurate quantum-chemical calculations may be helpful to predict or analyze unassigned CD spectra and can solve the above problems. The CD spectrum is theoretically obtained by calculating the excitation energies for peak positions and rotatory strengths for spectral intensities. The rotatory strength from the ground to excited states (*R_ge_*) in the dipole length representation is given by:

(1) Rge=Im[〈Ψg|μele|Ψe〉〈Ψe|μmag|Ψg〉]

where Ψ*_g_* and Ψ*_e_* are the electronic ground- and excited-state wavefunctions, and *μ_ele_* and *μ_mag_* are the electronic and magnetic transition dipole moment operators, respectively. So far, many CD calculations have been studied using random phase approximation (RPA) [2,3,4], time-dependent density functional theory (TDDFT) [5,6,7,8,9,10,11,12,13], and configuration interaction (CI) theory [14,15,16,17,18], for example, and these methods have reproduced experimental spectra qualitatively. However, the agreement of the calculated spectra with the experimental ones was not always satisfactory, and in some cases, even the signs of the CD intensities were opposite to the experimental peaks. Equation (1) indicates that the calculations should be performed accurately both on the ground and excited states in order to reproduce experimental CD spectra quantitatively. Therefore, to make a reliable theoretical analysis and assignment, one should use a more sophisticated theory. Symmetry adapted cluster (SAC) [19] and SAC-configuration interaction (SAC-CI) [20,21] methods are electron-correlation theories that can calculate ground and excited states with well-balanced accuracy. This theory has been applied to electronic studies for various systems [22] and has also been employed to obtain the CD spectra theoretically [23,24,25,26].

CD spectra for dichalcogenide compounds (ditellurides, diselenides, disulfides, and dioxides in some studies) have been studied experimentally [27,28] and theoretically [5,16,18,24] because of interest in the roles and stereochemistry of the chalcogen-chalcogen bonds in polypeptides, proteins, and other biochemical systems. We also carried out the SAC/SAC-CI calculations of CD spectra for twelve dichalcogenide molecules [24]. The calculated spectra were in good agreement with the corresponding experimental spectra, indicating that the SAC/SAC-CI method is very suitable for calculating CD spectra. In that article, we revealed that the differences in CD spectra among cyclic and acyclic dichalcogenide molecules can be understood in terms of the relationship between the dihedral angles and the molecular orbital energies, that the bands are red-shifted as the Ch atoms are replaced with heavier atoms, and so on.

The calculations of CD spectra for monochalcogenide compounds have not been reported as frequently, in spite of the fact that the experimental excitation and CD spectra were observed by Laur for cyclic and acyclic sulfides, selenides, and tellurides [28,29]. In the preceding studies, the band assignments were performed only for the bands in the UV spectra of sulfide and selenide molecules based on the semi-empirical calculations and the analogy with the sulfides, respectively. We recently carried out the SAC/SAC-CI calculations for cyclic monochalcogen compounds, (−)-(3a*S*,7a*S*)-2-chalcogena-*trans*-hydrindans, and reproduced their experimental UV and CD spectra and provided assignments to the observed bands [25]. In that study, we found that the first singlet excited states are assigned to n-σ* and the other states are assigned to n-Rydberg for all the calculated chalcogen compounds, differently from the previous assignments. On the other hand, for straight-chain monochalcogen molecules, the calculations of the CD spectra have not been reported as far as we know. The experimental excitation and CD spectra were observed by Laur for bis(2-methylbutyl)-chalcogenides, Ch[CH_2_CH(CH_3_)C_2_H_5_]_2_ (Ch = S, Se, Te) [28,29], but the band assignments were unconfirmed. One of the difficulties in the CD calculations for these systems is due to the flexibility of the molecule, namely, taking into consideration many conformation variations. The experimental CD intensities for bis(2-methylbutyl)chalcogenide were apparently small compared to other rigid molecules, and it can be attributed to the intensity cancellations among the conformations that have opposite intensities to each other. Furthermore, for bis(2-methylbutyl)telluride, Laur observed temperature dependence of the CD spectra. This indicates that the geometry interconversion among the conformations is restricted in the low temperature.

**Figure 1:**
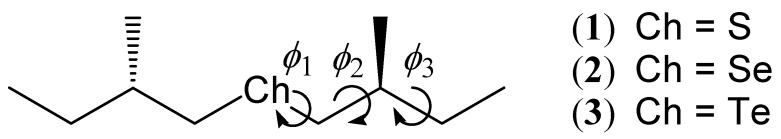
Molecular structures of the studied (+)-(*S*,*S*)-bis(2-methylbutyl)chalcogenides: (+)-(*S*,*S*)-bis(2-methylbutyl)sulfide (**1**), (+)-(*S*,*S*)-bis(2-methylbutyl)selenide (**2**) and (+)-(*S*,*S*)-bis(2-methylbutyl)telluride (**3**).

In this study, we carried out the SAC/SAC-CI calculations for (+)-(*S*,*S*)-bis(2-methylbutyl)sulfide (**1**), (+)-(*S*,*S*)-bis(2-methylbutyl)selenide (**2**), and (+)-(*S*,*S*)-bis(2-methylbutyl)telluride (**3**) (See Figure 1), and gave band assignments for the corresponding experimental UV and CD spectra. To consider the thermal effect, we considered 27 conformations for each molecule, and investigated their optimized molecular geometries and energies. The calculated CD spectrum for each stable conformation itself did not reproduce the experimental one, while the Boltzmann-averaged spectra showed good agreement with the experimental ones. Temperature dependence of the calculated spectra was also examined and is discussed in the latter section. 

## 2. Computational Details

All the molecular geometries were obtained by the MP2 optimization. Considering flexibility of the straight-chain compounds, we started optimizations from 27 initial geometries for each molecule, namely, three staggered conformation were treated for each bond rotation *ϕ*_1_, *ϕ*_2_, and *ϕ*_3_ in Figure 1, resulting in 3 × 3 × 3 = 27 conformations. Although we also considered the eclipsed conformations for the rotations *ϕ*_1–3_, the optimized conformations were converged to the staggered ones in every case. The basis sets used for the chalcogen (Ch = S, Se, and Te) atoms in the optimization calculations were the uncontracted [3s3p] LANL2DZ [30] augmented by the [2d] polarization functions (S: α_d_ = 0.659, 0.183; Se: α_d_ = 0.489, 0.144; Te: α_d_ = 0.305, 0.096) [31]. For the C and H atoms, the cc-pVDZ [32,33] and Huzinaga-Dunning double zeta (D95) with the standard scaling factors [34] basis sets were employed, respectively.

To calculate excitation and CD spectra of bis(2-methylbutyl)chalcogenides, twenty low-lying singlet excited states of the target molecules were calculated by SAC/SAC-CI SD-*R* method. For bis(2-methylbutyl)telluride, ten low-lying triplet excited states were also calculated. The perturbation selection procedures [35,36,37] were carried out with the thresholds in order to reduce the computational cost. The basis sets used for the Ch atoms in the SAC/SAC-CI calculations were the uncontracted [3s3p] LANL2DZ plus the [2d] polarization, [2s2p] diffuse, and [2s2p2d] Rydberg functions. (The polarization functions are the same as those in the previous paragraph. The exponents of the diffuse and Rydberg functions are as follows; α_s_^diff^ = 0.10792, 0.0426, α_p_^diff^ = 0.06593, 0.026025, α_s_^Ryd^ = 0.0437, 0.01725, α_p_^Ryd^ = 0.0380, 0.0150, α_d_^Ryd^ = 0.0285, 0.01125 for S; α_s_^diff^ = 0.0931988, 0.036789, α_p_^diff^ = 0.06232, 0.0246, α_s_^Ryd^ = 0.03121, 0.01232, α_p_^Ryd^ = 0.02714, 0.01071, α_d_^Ryd^ = 0.02036, 0.008036 for Se; α_s_^diff^ = 0.07163, 0.028275, α_p_^diff^ = 0.05206, 0.02055, α_s_^Ryd^ = 0.02230, 0.008801, α_p_^Ryd^ = 0.01939, 0.007653, α_d_^Ryd^ = 0.01454, 0.005740 for Te.) The aug-cc-pVDZ [32,33] and D95 basis sets were used for the C and H atoms, respectively. All the calculations were performed using a modified local version of the Gaussian 03 program package [38].

Theoretical excitation and CD spectral curves were drawn by the calculations of the excitation energies, oscillator strengths, and rotatory strengths of each excited state followed by fitting with normalized gaussian functions. The width of the fitting function is characterized by one standard deviation parameter, which was determined in order to reproduce the experimental spectral shapes. We used the dipole-length representation for the calculations of the transition moments and set the gauge origins at the gravity centers for each molecule. Although this representation generally has the gauge origin dependence for the CD calculations, we confirmed that this dependence was small for the molecules in this study.

## 3. Results and Discussion

### 3.1. Stable conformations for each ground-state molecule

First, we examined the stable conformations for bis(2-methylbutyl)sulfide (**1**). As explained in the preceding section, 27 conformations were considered as the initial geometries (*ϕ*_1_ = −60°, 60°, 180°, *ϕ*_2_ = −60°, 60°, 180°, and *ϕ*_3_ = −60°, 60°, 180°). Table 1 shows the initial and optimized values of *ϕ*_1–3_ and relative energies of their optimized geometries for the compound **1**. The Boltzmann weight values for each conformation at 298 K are also listed in the table. Generally speaking, the optimized angles are near to the initial values. In the case where the initial angle *ϕ*_1_ is set to 60°, the optimized angle can be a larger value (~100°). It is interpreted in terms of intramolecular steric repulsion between the two methyl groups in the 2-methylbutyl chains. This repulsion can be relaxed by an increase of the angle *ϕ*_1_.

**Figure 2:**
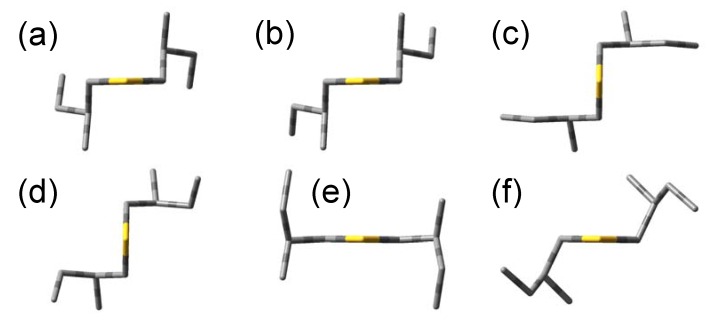
Most stable conformations of (+)-(*S*,*S*)-bis(2-methylbutyl)chalcogenides.

The optimized angles (*ϕ*_1_, *ϕ*_2_, *ϕ*_3_) = (57.9°, −174.6°, 66.8°) give the most stable conformation. This is derived from the initial angles (60°, 180°, 60°), and we call this conformation (a). Five conformations are found that have energy differences less than 1 kcal/mol to the most stable conformation (a), and we call them (b)–(f) in increasing order of energy. Since the other conformations have too high energies compared to (a)–(f), we ignore these conformations hereafter. Optimized molecular shapes of the sulfide compound for the conformations (a)–(f) are drawn in Figure 2. 

We also performed the corresponding geometry optimizations for bis(2-methylbutyl)selenide (**2**) and bis(2-methylbutyl)telluride (**3**). Similar to the sulfide, six conformations were calculated to be stable conformations for both compounds. The initial and optimized values of *ϕ*_1–3_ and relative energies of their optimized geometries for the six stable conformations of the compounds **1**–**3** are summarized in Table 2. These energy differences are within 1 kcal/mol, and therefore, we have to consider the conformations (a)–(f) as well as the most stable one in order to reproduce the excitation and CD spectra. The optimized values of *ϕ*_1–3_ are very similar among the three compounds, and the molecular shapes of the selenide and telluride compounds also resulted in almost the same as the sulfide in Figure 2.

### 3.2. Excitation and CD spectra of bis(2-methylbutyl)sulfide

**Figure 3:**
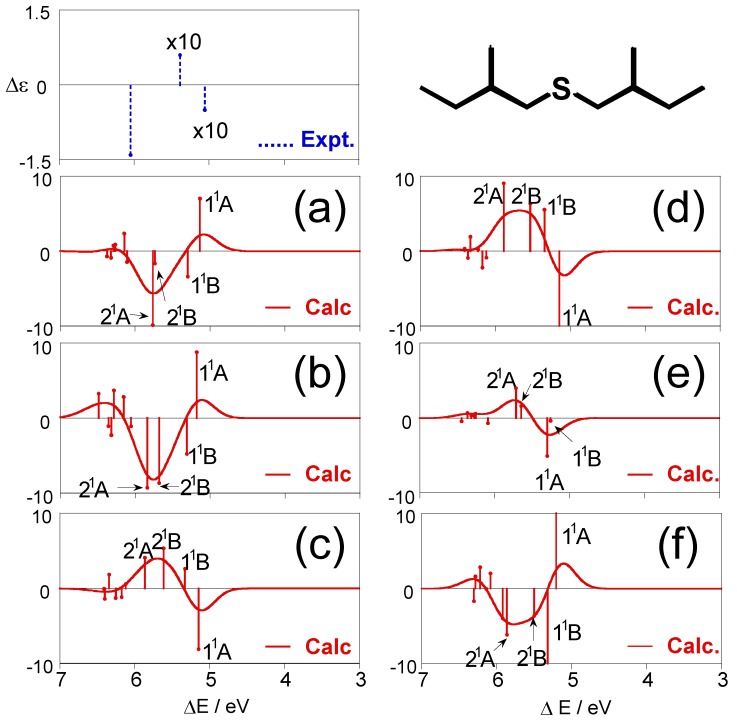
CD spectra calculated for each conformation of (+)-(*S*,*S*)-bis(2-methylbutyl)sulfide (**1**) together with the experimental spectrum [28].

Figure 3 shows individual CD spectra calculated for each conformation of (+)-(*S*,*S*)-bis(2-methylbutyl)sulfide (**1**), together with the experimental spectrum [28]. The CD spectra for the most stable conformation (a) obviously disagree with the experimental one, and the other conformations (b)–(f) also do not provide reproduction of the experimental results. One can see the similarity of the shapes of the CD spectra for (a) and (b), and (*ϕ*_1_, *ϕ*_2_, *ϕ*_3_) values are (57.9°, −174.6°, 66.8°) for (a) and (57.3°, −175.6°, 175.0°) for (b). For these conformations, the values of *ϕ*_1_ and *ϕ*_2_ are almost the same, and only *ϕ*_3_ values, the dihedral angles of the terminal methyl groups with respect to the principal chains, are largely different. The spectral shapes for (c) and (d) are also similar, and their (*ϕ*_1_, *ϕ*_2_, *ϕ*_3_) values are (−57.0°, −59.8°, 174.2°) and (−58.0°, −52.9°, −56.7°), respectively. Also for these conformations, only *ϕ*_3_ values are different from each other. These results indicate that the dihedral angle of the terminal methyl group does not affect the CD spectral shape so much.

**Figure 4:**
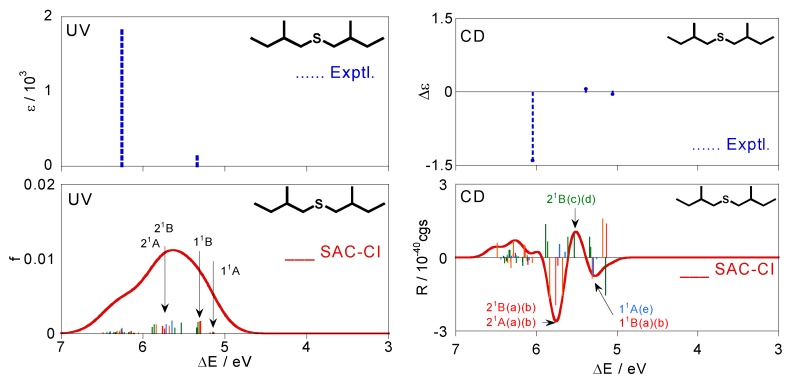
Excitation and CD spectra for (+)-(*S*,*S*)-bis(2-methylbutyl)sulfide (**1**) obtained by Boltzmann averaging of the calculated spectra (a)–(f) in Figure 3. The corresponding experimental spectra [28] are also shown.

The CD spectrum obtained by Boltzmann averaging the above six calculated spectra at 298 K is shown in Figure 4. In the experimental spectrum, negative, positive, and negative peaks are observed at 5.06, 5.39, and 6.05 eV, respectively. The calculated spectrum also exhibits positive, negative, and positive bands at ca. 5.30, 5.39, and 5.75 eV, respectively, and shows good agreement with the experimental trends. Since we reproduced the CD spectrum well, we analyze here the calculated spectrum. Four 1^1^A states of the conformations (a)–(d) exist at ca. 5.0 eV. However, these intensities offset each other and do not contribute to the total spectral shape consequently. The first negative band at *ca.* 5.3 eV is constructed by the negative 1^1^B peaks of (a) and (b), negative 1^1^A peak of (e), and positive 1^1^B peaks of (c) and (d). The 2^1^B states of (c) and (d) contribute to the second positive band at ca. 5.5 eV. The 2^1^B and 2^1^A states exist at around 5.75 eV for the many conformations. In particular, 2^1^B and 2^1^A of (a) and (b) have large negative intensities, and consequently, they predominantly contribute to the third-lowest intense negative band. On the other hand, comparison between the experimental and calculated excitation spectra is somewhat difficult. Two bands are observed at 5.34 and 6.26 eV in the experimental UV spectrum. In the calculated spectrum, the 1^1^B states of the six conformations (a)–(f) are found at ca. 5.3 eV, and many peaks derived from the 2^1^B and 2^1^A states of all the conformations form a band at ca. 5.7 eV. We assigned these states to the two experimental bands. The second band at 6.26 eV in the experimental spectrum is reported as a shoulder peak, indicating that more intense bands are observed at the higher energy region. This might be reproducible theoretically by augmenting the number of excited states to calculate, although such a calculation was not performed in this study. Excitation energies *ΔE*, oscillator strengths *f*, rotatory strengths *R*, second moments −*e*<*r*^2^>, and electronic structures of the excited states for the sulfide compound **1** are summarized in Table 3, together with the experimental data, namely, excitation energy *ΔE*, molar absorption coefficient *ε*, and the difference between *ε* of left- and right-hand circularly polarized lights *Δε*. Correspondences between the calculated states and the experimental bands are also shown in the table.

For example, the vertical line beside the experimental UV band at 5.34 eV in Table 3 means that this band consists of the six states of 1^1^B(a)–1^1^B(e) according to our assignment. Every excited state has one-electron excitations from the #44 MO as main configurations, which is the HOMO of this molecule. The main character of the HOMO is lone pairs (n) on the chalcogen atom. We compared the second moments of the excited states to that of the ground state in order to determine the nature of each state. The lowest excited state, 1^1^A, has a second moment value close to the ground-state one for every conformation, and therefore, we assigned this state to n-σ*. The other excited states were assigned to n-Rydberg excitations judging from the larger second moments of these states.

### 3.3. Excitation and CD spectra of bis(2-methylbutyl)selenide

**Figure 5:**
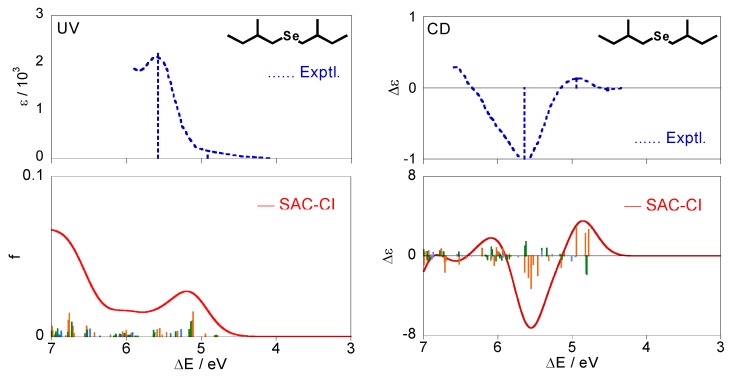
Excitation and CD spectra for (+)-(*S*,*S*)-bis(2-methylbutyl)selenide (**2**) obtained by Boltzmann averaging of the calculated spectra for the six conformations. The corresponding experimental spectra [28] are also shown.

Figure 5 shows the calculated excitation and CD spectra of (+)-(*S*,*S*)-bis(2-methylbutyl)selenide (**2**) together with the experimental spectra [28]. The calculated spectra were obtained by Boltzmann averaging of the conformations (a)–(f) for (**2**) at 298 K. Like compound **1**, the individual CD spectra for each conformation disagree with the experimental one, whereas the averaged spectrum were in agreement. 

Table 4 shows details of electronic states for the selenide compound **2**. All the states are described by excitations from the HOMO (#44) with the n character. The lowest excited state is 1^1^A except for (e) and is assigned to n-σ*. The other states are assigned to n-Rydberg in view of their second moments. For the CD spectra in Figure 5, several states with mainly positive intensities were calculated at ca. 4.8 eV, and reproduced a positive band at 4.94 eV in the experimental spectrum. These are associated with the n-σ* 1^1^A state for each conformer, as seen from Table 4. One can observe intense negative peaks at ca. 5.6 eV both in the experimental and theoretical spectra. This peak is mainly assigned to the 2^1^A states for the conformations (a)(b)(f), and the 2^1^B states for (a)(b) also contribute to the band intensity slightly. For the excitation spectra, the weak band is observed at 4.92 eV in the experiments and corresponds to the 1^1^A states for all the conformers similar to the CD spectrum. On the other hand, the interpretation of the intense band at 5.58 eV is somewhat difficult. We have a spectral peak at ca. 5.2 eV and a broad band at the higher energy region in our calculations. One can associate the peak at 5.2 eV with the experimental one at 5.58 eV, and according to this interpretation, this peak is mainly assigned to the 1^1^B states. However, this interpretation is rather unnatural considering that the peak at 5.64 eV in the experimental CD spectrum was mainly assigned to 2^1^A. Therefore, we suggest that the peak at 5.58 eV in the experimental UV spectrum is constructed by many electronic states on 5–6 eV, and it is assigned to the 1^1^B, 2^1^B, and 2^1^A states (n-Rydberg excitation). Introduction of the effect of the vibrational states can improve the intensities in the calculated UV spectrum although it is not examined in this study.

Although a very weak negative band is observed at 4.52 eV in the experimental CD spectrum, we can find no (singlet) electronic states in this energy region. It can be attributed to triplet excited states through the spin-orbit interaction. 

### 3.4. Excitation and CD spectra of bis(2-methylbutyl)telluride

**Figure 6:**
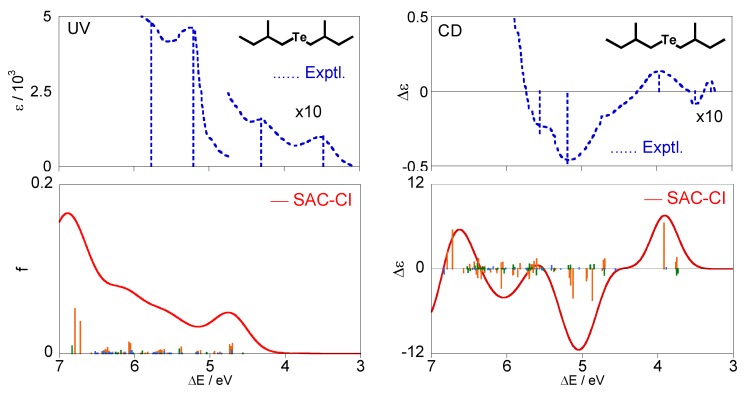
Excitation and CD spectra for (+)-(*S*,*S*)-bis(2-methylbutyl)telluride (**3**) obtained by Boltzmann averaging of the calculated spectra for the six conformations. The corresponding experimental spectra [28] are also shown.

The calculated excitation and CD spectra of (+)-(*S*,*S*)-bis(2-methylbutyl)telluride (**3**) together with the experimental spectra [28] are shown in Figure 6 The calculated spectra were obtained by Boltzmann averaging of the six conformations at 298 K, as in the cases of compounds **1** and **2**. Our calculation results reproduced their experimental spectral shapes on the whole.

Details of electronic states for the telluride compound **3** are summarized in Table 5. Also for this molecule, all the excited states are described by electron promotions from the HOMO (#44) with the n character. The lowest excited state for each conformation is 1^1^A and is assigned to n-σ*. The other states are assigned to n-Rydberg judging from their second moments. 

One can see experimental CD bands at 3.28(+, weak), 3.49(−, weak), 3.97(+), 5.19(−, intense), and 5.56 eV (−, shoulder) in Figure 6. Our calculations reproduce well the third and fourth bands at 3.97 and 5.19 eV, respectively. The corresponding bands were calculated at ca. 3.9 eV and 5.1 eV and were assigned mainly to 1^1^A for the conformation (f) (n-σ*) and 2^1^A and 2^1^B for (f)(a)(b) (n-Rydberg), respectively. However, we could not reproduce the other experimental bands in the present calculations. The very weak peaks are observed at 3.28 and 3.49 eV, whereas no (singlet) states were calculated in this energy region. They are probably associated with the triplet excitations because the lowest triplet state 3^1^A was found at 3.70 eV in our calculations. Also for the shoulder band at 5.56 eV, the corresponding bands did not appear in the calculated spectrum. This disharmony could be attributed to triplet excitations and/or insufficiency of calculation accuracy including consideration of the vibronic coupling, and therefore, more accurate calculations are required for conclusive analyses and assignments on this molecule. In the experimental excitation spectrum, two weak bands are observed at 3.48 and 4.31 eV, and two intense bands are found at 5.21 and 5.77 eV. The first peak at 3.48 eV corresponds to the CD bands at3.28 and 3.49 eV and is expected to be due to triplet excitations. The second peak at 4.31 eV is assigned to the n-σ* 1^1^A states for all the conformers, which can correspond to the positive band at 3.97 eV in the CD spectrum. For the intense band at 5.21 and 5.77 eV, we have a broad band at the energy region higher than ca. 4.7 eV in our calculations and suggest that the intense bands in the experimental UV spectrum are constructed by many electronic states, similar to compound **2**. They are assigned to the 1^1^B, 2^1^B, and 2^1^A states for all the conformation (n-Rydberg excitation).

**Figure 7:**
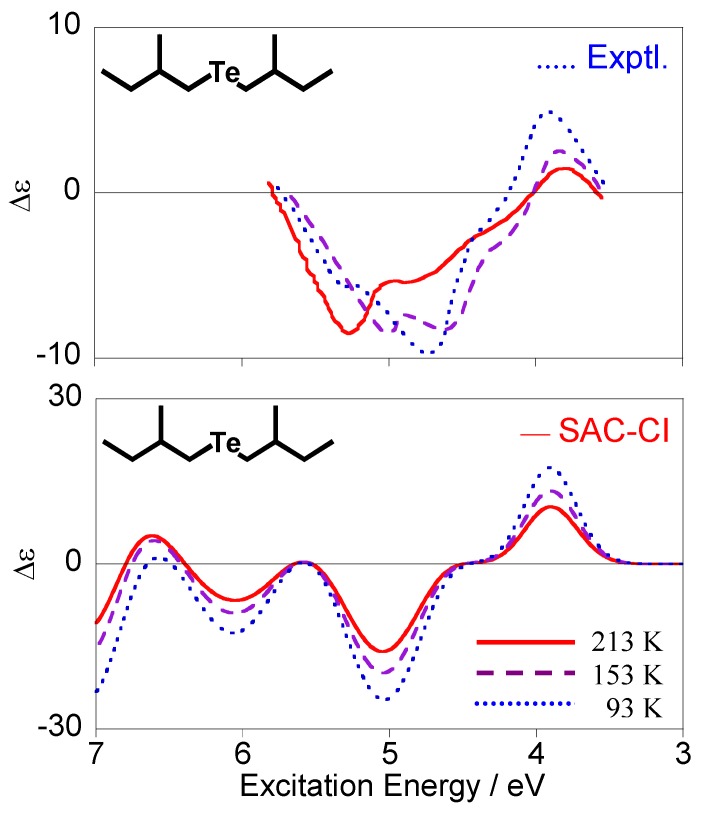
Temperature dependence of the observed (upper) [28] and calculated (lower) CD spectra for (+)-(*S*,*S*)-bis(2-methylbutyl)telluride (**3**).

Since Boltzmann weight factors are dependent on temperature, the ratio among the conformations is also changed by the temperature. Therefore, if temperature dependence of the spectra is observed, we can calculate and compare it with the experimental one. Figure 7 shows the experimental [28] and calculated CD spectra of compound **3** at 93, 153, and 213 K. Our calculations reproduced the observed spectral change qualitatively. Generally speaking, increases in temperature will reduce CD intensities because intensity cancellation can easily occur among the conformations with the opposite intensities. The CD spectra in Figure 7 also exhibit this tendency on the whole, in particular for the calculated spectra. In the experimental spectra, the most intense wavelength is dependent on the temperature, while no dependence was observed in the calculated ones. Although the origin of this feature is unknown at present, we speculate that it can be associated with the conformation dependence of the triplet excitation energy for the n-Rydberg states.

## 4. Conclusions

Theoretical electronic excitation and natural circular dichroism (CD) spectra of (+)-(*S*,*S*)-bis(2-methylbutyl)chalcogenide, Ch[CH_2_CH(CH_3_)C_2_H_5_]_2_ (Ch = S, Se, Te), were calculated by the symmetry adapted cluster (SAC) and SAC-configuration interaction (SAC-CI) methods. These compounds have six stable conformers, and the Boltzmann-averaged spectra for those conformers showed good agreement with the experimental results. The lowest bands were assigned to n-σ* and the others were to n-Rydberg for the compounds **1**–**3** in common. For the telluride compound **3**, some spectral characteristics were not reproduced in our calculations, namely, weak bands at ca. 3.4 eV, a shoulder peak at ca. 5.5 eV, and temperature dependence of the band at ca. 5.0 eV. The bands at ca. 3.4 eV can be attributed to triplet transitions through the spin-orbit interaction effects. For the bands at ca. 5 eV, triplet excitations and/or accuracy insufficiency would cause lacks of the bands in our calculations, and further investigations are necessary for conclusive discussions.

## Figures and Tables

**Table 1:** Optimizations of the dihedral angles for (+)-(*S*,*S*)-bis(2-methylbutyl)sulfide (**1**). The angles *ϕ*_1–3_ stand for the dihedral angles with respect to the rotations around the bonds in Figure 1. The shapes of the conformations (a)–(f) are drawn in Figure 2.

Initial angles [deg]				Optimized angles [deg]				Relative energy [kcal/mol]	Boltzmann weight (at 298K)	
*ϕ*_1_	*ϕ*_2_	*ϕ*_3_		*ϕ*_1_	*ϕ*_2_	*ϕ*_3_				
60	60	60		98.7	58.4	55.6		0.727	0.057	(f)
60	60	180		98.5	63.2	172.9		1.684	0.011	
60	60	−60		98.0	77.7	−66.9		6.347	0.000	
60	180	60		57.9	−174.6	66.8		0.000	0.195	(a)
60	180	180		57.3	−175.6	175.0		0.052	0.179	(b)
60	180	−60		56.9	−172.0	-62.1		1.171	0.027	
60	−60	60		94.0	−67.1	87.2		6.536	0.000	
60	−60	180		98.7	−62.8	176.3		1.862	0.008	
60	−60	−60		98.1	−67.5	−69.9		2.836	0.002	
180	60	60		176.7	57.4	53.9		0.224	0.134	(e)
180	60	180		178.5	63.6	171.1		1.877	0.008	
180	60	−60		171.9	71.3	−77.8		5.661	0.000	
180	180	60		172.7	−177.3	61.8		1.849	0.009	
180	180	180		177.1	−171.6	175.7		1.786	0.010	
180	180	−60		180.0	−167.1	−61.0		2.584	0.002	
180	−60	60		−171.5	−84.9	62.6		5.047	0.000	
180	−60	180		−174.7	−63.2	176.5		1.805	0.009	
180	−60	−60		−174.6	−56.1	−54.9		1.341	0.020	
−60	60	60		−69.6	71.6	54.7		24.901	0.000	
−60	60	180		−98.2	64.5	174.0		1.617	0.013	
−60	60	−60		−83.6	103.1	−71.0		13.944	0.000	
−60	180	60		−97.3	−175.2	62.5		2.324	0.004	
−60	180	180		−98.6	−172.9	174.5		2.018	0.006	
−60	180	−60		−98.0	−169.1	−63.6		2.913	0.001	
−60	−60	60		−53.2	−74.3	72.0		4.837	0.000	
−60	−60	180		−57.0	−59.8	174.2		0.136	0.155	(c)
−60	−60	−60		−58.0	−52.9	−56.7		0.162	0.149	(d)

**Table 2:** Summary of optimizations and relative energies for (+)-(*S*,*S*)-bis(2-methylbutyl)chalcogenides. Definitions of the angles *ϕ*_1–3_ are the same as Table 1. The shapes of the conformations (a)–(f) are drawn in Figure 2.

Compound	Initial angles [deg]				Optimized angles [deg]			Relative energy [kcal/mol]	Boltzmann weight (at 298K)	
	*ϕ*_1_	*ϕ*_2_	*ϕ*_3_		*ϕ*_1_	*ϕ*_2_	*ϕ*_3_			
Ch = S	60	180	60		57.9	−174.6	66.8	0.000	0.195	(a)
	60	180	180		57.3	−175.6	175.0	0.052	0.179	(b)
	−60	−60	180		−57.0	−59.8	174.2	0.136	0.155	(c)
	−60	−60	−60		−58.0	−52.9	−56.7	0.162	0.149	(d)
	180	60	60		176.7	57.4	53.9	0.224	0.134	(e)
	60	60	60		98.7	58.4	55.6	0.727	0.057	(f)
										
Ch = Se	60	180	60		57.4	−172.5	66.8	0.000	0.233	(a)
	60	180	180		56.3	−172.4	175.5	0.160	0.178	(b)
	−60	−60	180		−56.1	−63.1	173.9	0.310	0.138	(c)
	−60	−60	−60		−56.8	−54.9	−56.3	0.427	0.113	(d)
	60	60	60		97.7	58.4	54.8	0.499	0.100	(f)
	180	60	60		174.1	56.8	54.1	0.671	0.075	(e)
										
Ch = Te	60	60	60		95.9	58.8	54.3	0.000	0.232	(f)
	60	180	60		56.3	−170.3	66.2	0.106	0.194	(a)
	60	180	180		54.6	−167.8	175.9	0.339	0.131	(b)
	−60	−60	180		−54.8	−67.9	173.5	0.542	0.093	(c)
	−60	−60	−60		−55.2	−58.0	−55.8	0.773	0.063	(d)
	180	60	60		167.4	56.1	54.9	0.837	0.056	(e)

**Table 3:** Excitation energies *ΔE* (in eV), oscillator strengths *f*, rotatory strengths *R* (in 10^−40^ cgs unit), second moments −*e*<*r*^2^> (in au), and electronic structures of the excited states calculated for all the conformations (a)–(f) of (+)-(*S*,*S*)-bis(2-methylbutyl)sulfide (**1**).

SAC-CI								Experiments	
State*^a^*	Main configurations (|c|>0.4)	Nature	*ΔE*	*f*	*R*	−*e*<*r*^2^>		*ΔE*(*ε*)*^b^*	*ΔE*(*Δε*)
1^1^A (a)	0.57(44-46)-0.52(44-57)-0.47(44-80)	n-σ*	5.14	0.0006	7.04	−219.21			
1^1^A (d)	0.60(44-46)-0.40(44-55)-0.40(44-81)	n-σ*	5.15	0.0006	−8.11	−217.48			5.06(−0.05)
1^1^A (e)	0.60(44-45)-0.40(44-54)	n-σ*	5.15	0.0013	−10.33	−216.65			
1^1^A (b)	0.59(44-46)-0.51(44-57)-0.43(44-79)	n-σ*	5.18	0.0007	8.81	−220.17			
1^1^A (f)	0.64(44-45)-0.45(44-55)	n-σ*	5.21	0.0042	32.65	−218.11			
1^1^B (c)	0.78(44-45)	n-Ryd(p_z_)	5.26	0.0001	−0.37	−237.69			
1^1^B (a)	0.78(44-45)	n-Ryd(p_z_)	5.30	0.0083	−3.39	−261.84		*5.34*(170)	
1^1^A (c)	0.47(44-77)	n-σ*	5.30	0.0002	−5.08	−200.37			
1^1^B (b)	0.73(44-45)	n-Ryd(p_z_)	5.31	0.0089	−4.78	−257.90			
1^1^B (f)	0.69(44-47)+0.47(44-53)	n-Ryd(p_z_)	5.32	0.0061	−23.36	−242.12			
1^1^B (d)	0.74(44-45)	n-Ryd(p_z_)	5.33	0.0097	2.62	−256.91			
1^1^B (e)	0.59(44-47)-0.54(44-46)	n-Ryd(p_z_)	5.35	0.0050	5.55	−247.79			
2^1^B (f)	0.79(44-46)	n-Ryd(s)	5.50	0.0078	−7.24	−252.23			
2^1^B (e)	0.61(44-46)+0.53(44-47)-0.42(44-56)	n-Ryd(s)	5.53	0.0094	6.37	−246.23			5.39(+0.06)
2^1^B (d)	0.64(44-48)	n-Ryd(s)	5.62	0.0047	5.33	−241.83			
2^1^B (c)	0.71(44-47)	n-Ryd(s)	5.65	0.0126	1.58	−242.14			
2^1^B (b)	0.62(44-48)-0.40(44-53)-0.39(44-45)	n-Ryd(s)	5.68	0.0056	−8.65	−245.20			
2^1^A (c)	0.79(44-46)	n-Ryd(p_xy_)	5.72	0.0090	3.98	−258.59			
2^1^B (a)	0.60(44-48)-0.51(44-53)	n-Ryd(s)	5.74	0.0031	−1.62	−253.72			
2^1^A (a)	0.78(44-47)	n-Ryd(p_xy_)	5.76	0.0050	−9.85	−270.95		*6.26*(1850)	6.05(−1.40)
2^1^A (b)	0.78(44-47)	n-Ryd(p_xy_)	5.84	0.0064	−9.29	−273.38			
2^1^A (d)	0.81(44-47)	n-Ryd(p_xy_)	5.86	0.0076	4.08	−275.85			
2^1^A (f)	0.49(44-48)-0.49(44-45)	n-Ryd(p_xy_)	5.87	0.0010	−12.30	−253.23			
2^1^A (e)	0.70(44-48)+0.47(44-45)	n-Ryd(p_xy_)	5.89	0.0055	9.08	−271.98			

*^a^* Characters in the parentheses stand for the conformation indices;

*^b^* Italic values stand for shoulder peaks.

**Table 4:** Excitation energies *ΔE* (in eV), oscillator strengths *f*, rotatory strengths *R* (in 10^−40^ cgs unit), second moments −*e*<*r*^2^> (in au), and electronic structures of the excited states calculated for all the conformations (a)–(f) of (+)-(*S*,*S*)-bis(2-methylbutyl)selenide (**2**).

SAC-CI								Experiments	
State *^a^*	Main configurations (|c|>0.4)	Nature	*ΔE*	*f*	*R*	−*e*<*r*^2^>		*ΔE*(*ε*) *^b^*	*ΔE*(*Δε*)
1^1^A (a)	0.57(44-54)+0.55(44-72)-0.41(44-45)	n-σ*	4.78	0.0009	28.48	−215.39		*4.92*(140)	4.94(+0.13)
1^1^A (d)	0.57(44-54)-0.46(44-45)+0.42(44-73)	n-σ*	4.80	0.0025	−41.14	−213.95			
1^1^A (c)	0.58(44-54)-0.51(44-73)-0.46(44-45)	n-σ*	4.81	0.0011	−31.28	−215.51			
1^1^A (b)	0.58(44-54)+0.50(44-73)-0.44(44-45)	n-σ*	4.82	0.0012	32.16	−217.17			
1^1^A (f)	0.51(44-55)-0.48(44-45)+0.40(44-73)	n-σ*	4.94	0.0073	70.37	−213.65			
1^1^B (e)	0.72(44-45)	n-Ryd(p_z_)	4.96	0.0006	−1.40	−245.28			
1^1^A (e)	0.56(44-74)	n-σ*	5.01	0.0002	−15.80	−203.06			
1^1^B (a)	0.69(44-46)	n-Ryd(p_z_)	5.11	0.0328	2.49	−264.46		*5.58*(2250)	
1^1^B (b)	0.65(44-46)+0.43(44-53)	n-Ryd(p_z_)	5.12	0.0281	0.28	−263.14			
1^1^B (c)	0.64(44-46)	n-Ryd(p_z_)	5.14	0.0334	−6.81	−260.92			
1^1^B (f)	0.61(44-47)-0.49(44-53)	n-Ryd(s)	5.15	0.0172	−28.69	−252.52			
1^1^B (d)	0.63(44-47)+0.40(44-53)	n-Ryd(p_z_)	5.16	0.0195	3.71	−256.16			
2^1^B (e)	0.58(44-48)+0.43(44-54)	n-Ryd(s)	5.27	0.0280	4.47	−243.12			
2^1^B (f)	0.71(44-46)-0.51(44-54)	n-Ryd(p_z_)	5.32	0.0145	−11.57	−255.00			
2^1^B (d)	0.61(44-46)-0.50(44-55)	n-Ryd(s)	5.34	0.0124	17.07	−253.21			
2^1^B (c)	0.56(44-48)+0.42(44-53)+0.41(44-55)	n-Ryd(s)	5.41	0.0042	13.46	−252.77			
2^1^A (e)	0.64(44-46)-0.49(44-56)+0.44(44-47)	n-Ryd(p_xy_)	5.41	0.0107	16.90	−266.72			
2^1^B (b)	0.52(44-48)-0.48(44-55)-0.45(44-53)	n-Ryd(s)	5.46	0.0068	−27.91	−255.66			5.64(−1.1)
2^1^B (a)	0.55(44-53)+0.48(44-48)+0.42(44-55)	n-Ryd(s)	5.52	0.0016	−9.03	−261.24			
2^1^A (a)	0.67(44-47)+0.44(44-45)-0.40(44-56)	n-Ryd(p_xy_)	5.55	0.0107	−34.88	−279.68			
2^1^A (b)	0.66(44-47)+0.46(44-45)	n-Ryd(p_xy_)	5.59	0.0117	−30.13	−280.76			
2^1^A (d)	0.57(44-48)-0.53(44-45)	n-Ryd(p_xy_)	5.62	0.0096	31.61	−283.49			
2^1^A (c)	0.72(44-47)+0.41(44-56)	n-Ryd(p_xy_)	5.64	0.0140	17.82	−283.72			
2^1^A (f)	0.66(44-45)	n-Ryd(p_xy_)	5.64	0.0040	−40.63	−261.18			

*^a^* Characters in the parentheses stand for the conformation indices.

*^b^* Italic values stand for shoulder peaks.

**Table 5:** Excitation energies *ΔE* (in eV), oscillator strengths *f*, rotatory strengths *R* (in 10^−40^ cgs unit), second moments −*e*<*r*^2^> (in au), and electronic structures of the excited states calculated for all the conformations (a)–(f) of (+)-(*S*,*S*)-bis(2-methylbutyl)telluride (**3**).

SAC-CI								Experiments	
State*^a^*	Main configurations (|c|>0.4)	Nature	*ΔE*	*f*	*R*	−*e*<*r*^2^>		*ΔE*(*ε*)*^b^*	*ΔE*(*Δε*)
1^1^A (d)	0.55(44-66)-0.52(44-54)	n-σ*	3.73	0.0006	−41.43	−209.26		4.31(160)	
1^1^A (a)	0.56(44-67)+0.53(44-54)	n-σ*	3.75	0.0002	20.45	−211.68			
1^1^A (c)	0.64(44-67)-0.53(44-54)	n-σ*	3.75	0.0003	−21.90	−210.83			
1^1^A (b)	0.69(44-67)+0.53(44-54)	n-σ*	3.76	0.0002	17.56	−211.81			
1^1^A (e)	0.74(44-67)	n-σ*	3.89	0.0000	8.91	−201.61			
1^1^A (f)	0.67(44-66)	n-σ*	3.92	0.0025	70.25	−205.00			3.97(+0.13)
1^1^B (e)	0.61(44-45)-0.53(44-54)	n-Ryd(s)	4.56	0.0058	−6.85	−248.11			
1^1^B (a)	0.60(44-46)-0.48(44-55)	n-Ryd(p_z_)	4.70	0.0608	18.29	−270.91		5.21(4600)	
2^1^B (e)	0.51(44-48)-0.40(44-62)	n-Ryd(p_z_)	4.71	0.0467	3.96	−249.24			
1^1^B (d)	0.51(44-47)-0.49(44-55)	n-Ryd(p_z_)	4.71	0.0537	−13.81	−262.71			
1^1^B (c)	0.57(44-46)-0.50(44-55)	n-Ryd(p_z_)	4.71	0.0645	−22.54	−266.70			
1^1^B (b)	0.61(44-46)-0.43(44-55)	n-Ryd(p_z_)	4.72	0.0629	23.57	−271.86			
1^1^B (f)	0.59(44-47)+0.43(44-55)	n-Ryd(s)	4.74	0.0451	−1.62	−253.90			
2^1^B (d)	0.52(44-47)	n-Ryd(s)	4.85	0.0056	33.54	−257.85			
2^1^B (f)	0.65(44-45)-0.44(44-55)	n-Ryd(p_z_)	4.87	0.0063	−47.96	−264.49			5.19(−0.48)
2^1^B (c)	0.57(44-47)	n-Ryd(s)	4.90	0.0099	15.20	−253.02			
2^1^B (a)	0.51(44-48)-0.40(44-71)-0.39(44-55)	n-Ryd(s)	4.94	0.0116	−15.70	−253.73			
2^1^B (b)	0.51(44-48)-0.45(44-55)-0.42(44-70)	n-Ryd(s)	4.95	0.0075	−31.50	−249.63			
2^1^A (e)	0.53(44-46)+0.52(44-55)-0.45(44-47)	n-Ryd(p_xy_)	5.05	0.0103	6.78	−282.66			
2^1^A (f)	0.69(44-46)-0.44(44-54)	n-Ryd(p_xy_)	5.12	0.0144	−44.02	−285.02			
2^1^A (a)	0.51(44-45)-0.51(44-47)+0.46(44-56)	n-Ryd(p_xy_)	5.16	0.0138	−28.89	−288.98			
2^1^A (d)	0.65(44-45)	n-Ryd(p_xy_)	5.16	0.0145	30.47	−290.54			
2^1^A (b)	0.58(44-45)+0.47(44-56)-0.42(44-47)	n-Ryd(p_xy_)	5.17	0.0173	−22.83	−285.36			
2^1^A (c)	0.54(44-45)+0.47(44-48)-0.44(44-56)	n-Ryd(p_xy_)	5.19	0.0160	12.21	−287.75			

*^a^* Characters in the parentheses stand for the conformation indices.

*^b^* Italic values stand for shoulder peaks.
